# Misdiagnosis of multiple synchronous small bowel adenocarcinomas as intestinal tuberculosis: a case report

**DOI:** 10.1186/s12876-020-01271-6

**Published:** 2020-04-16

**Authors:** Qiwei Li, Tao Chen, Hexi Cui, Xiao Xiao, Chunqiu Chen, Zhenyu Shen, Fu Ji, Lu Yin

**Affiliations:** 1 Department of General Surgery, South Campus, Renji Hospital, School of Medicine, Shanghai Jiao Tong University, Shanghai, 200112 China; 2 Department of Nursing, South Campus, Renji Hospital, School of Medicine, Shanghai Jiao Tong University, Shanghai, 200112 China; 3 Department of Tuberculosis Control, Shanghai Municipal Center for Disease Control and Prevention, Shanghai, 200336 China; 4 Department of Abdominal Surgery, Shanghai Tenth People’s Hospital, Tongji University School of Medicine, Shanghai, 200072 China; 5 Department of Gastrointestinal Surgery, Renji Hospital, School of Medicine, Shanghai Jiao Tong University, Shanghai, 200127 China

**Keywords:** Small bowel cancer, Small bowel adenocarcinoma, Intestinal tuberculosis, Diagnosis, High-risk

## Abstract

**Background:**

Small bowel adenocarcinoma (SBA) is a rare malignancy that primarily occurs in the duodenum. Multiple synchronous SBA is unique rare and difficult to diagnose due to non-specific disease presentation. Protocols to identify multiple synchronous SBA during early disease stages are urgently required.

**Case presentation:**

An elderly man experienced left lower abdominal pain and melena for 3 months. Abdominal CT showed thickening of the multiple segmental small intestinal walls. As the patient had pulmonary tuberculosis simultaneously, he was misdiagnosis as intestinal tuberculosis and received anti-spasm therapy. The treatment delayed radical resection surgery and the patient underwent palliative segmental resection of the jejunum after 4 months due to intestinal obstruction. Resected specimens showed multiple synchronous SBA (five tumors). The patient accepted chemotherapy postoperatively. Six months postoperatively, the patient died of brain metastasis.

**Conclusions:**

We highlight how multiple synchronous SBA is rare and easily misdiagnosed. We should rule out multiple synchronous SBA when diagnosing intestinal diseases (e.g. inflammatory bowel disease, IBS). Intestinal tuberculosis may also be one of the risk factors for multiple synchronous SBA. High-risk patients should be assessed for known tumor makers, and receive gastroscopy, enteroscopy or capsule endoscopy. Doctors should obtain the pathology under endoscopy to the greatest possible degree. For suspected patients, laparotomy should be performed.

## Background

Small bowel cancer is a rare malignancy that comprises less than 5% of all gastrointestinal malignancies [1]. Its annual incidence is approximately 13.9 cases per million individuals. Small bowel cancer has four common histological types: adenocarcinoma (30–40%), carcinoid tumor (35–42%), lymphoma (15–20%), and sarcoma (10–15%) [2]. Small bowel adenocarcinoma (SBA) is most commonly located in the duodenum (57%), 29% of cases are located in the jejunum, and 10% of cases are in the ileum [3]. The clinical presentation of SBA is non-specific abdominal discomfort, including abdominal pain, nausea, vomiting, gastrointestinal bleeding and intestinal obstruction. This leads to an average delay of 6–10 months in diagnosis [4].

The majority of SBA are single with multiple synchronous SBA rare. Due this rarity, multiple synchronous SBA is frequently misdiagnosed and knowledge on the clinical characteristics, treatment modalities, and prognosis of patients is sparse, particularly in the Asian community.

In this study, we report a case of multiple synchronous SBA in the jejunum in a 70-year-old man. The patient was misdiagnosed as intestinal tuberculosis and accepted anti-tuberculosis treatment until bowel obstruction with lymph node metastasis was observed. Palliative segmental bowel resection was performed. The patient died postoperatively after 6 months. This highlights how multiple synchronous SBA is rare and easily misdiagnosed during early stages.

## Case presentation

**Fig. 1:**
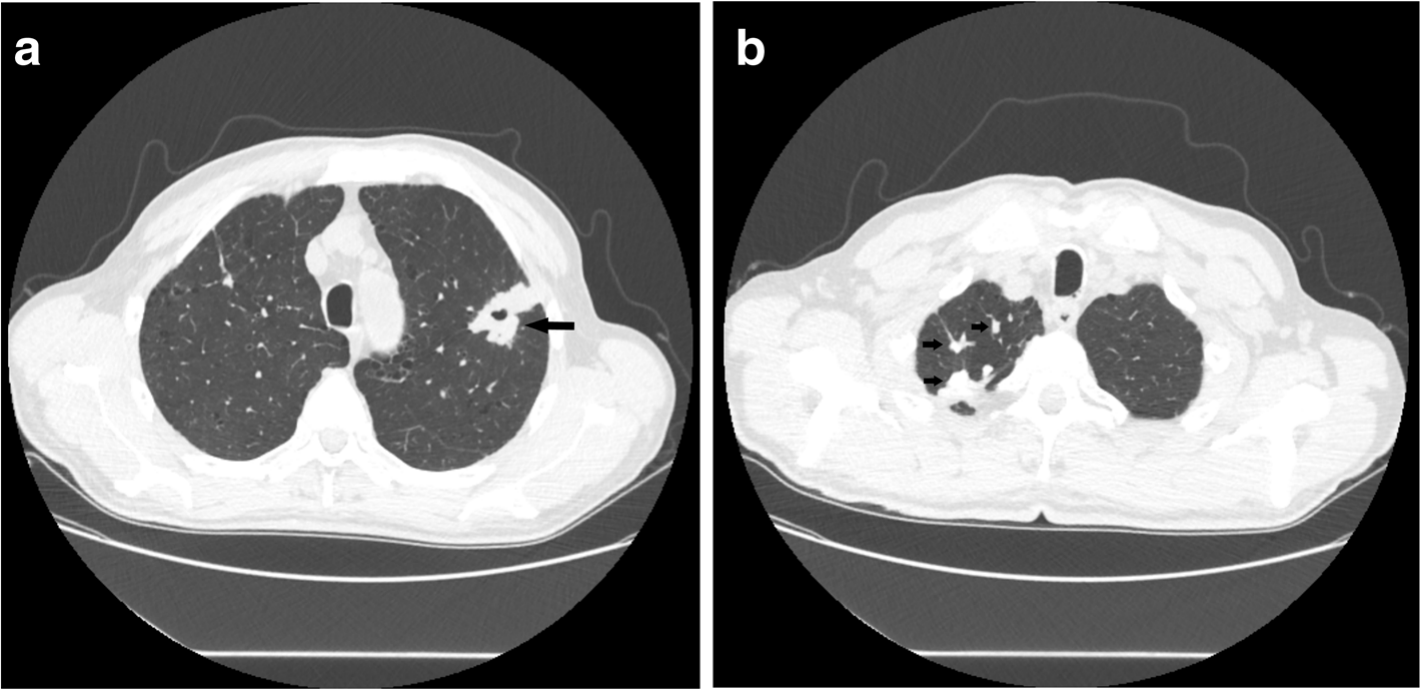
Pulmonary CT. **a** A cavity lesion (black arrow) in the posterior segment of the upper lobe of the left lung. **b** Multiple nodules and spots (black arrow) in the upper lobe of the right lung

The patient was a 70-year-old male who had experienced left lower abdominal pain and melena for 3 months. Physical examination revealed a soft abdomen with tenderness in the left lower quadrant. A mass of 5 cm in diameter was identified. The mass boundary was unclear and the mass was immobile. The patient had no family history. Laboratory tests included fecal occult blood ++; hemoglobin 60 g/liter; interferon-gamma release assays for *Mycobacterium tuberculosis* (TB-IGRA) +; T spot +; sputum culture: no pathogenic bacteria growth; sputum smear: no acid-fast bacilli; anti-tuberculosis antibodies IgG -; anti-tuberculosis antibodies IgM -; erythrocyte sedimentation rate (ESR) 50 mm/h; Ca211: 12.4 ng/ml, Ca125 and Ca153 were normal. SCC: 5.3 ng/ml; anti-streptolysin (ASO): 133 IU/ml; rheumatoid factor (RF): 17 IU/ml. Pulmonary CT (Fig. 1a-b) identified a cavity lesion in the posterior segment of the upper lobe of the left lung, enlarged lymph nodes in the left hilar and mediastinum, emphysema, multiple pulmonary bullae, and posterior segmental tuberculosis. Abdominal CT identified uneven thickening of the small intestine with localized dilatation in the left middle abdomen and multiple retroperitoneal lymph nodes. Gastroscopy showed no abnormalities, but colonoscopy revealed multiple polyps in colon and rectum. Initially, the patient was diagnosed with secondary pulmonary tuberculosis and intestinal tuberculosis in the infectious disease hospital. After 2 weeks of HERZ treatment, the abdominal pain did not alleviate.

**Fig. 2:**
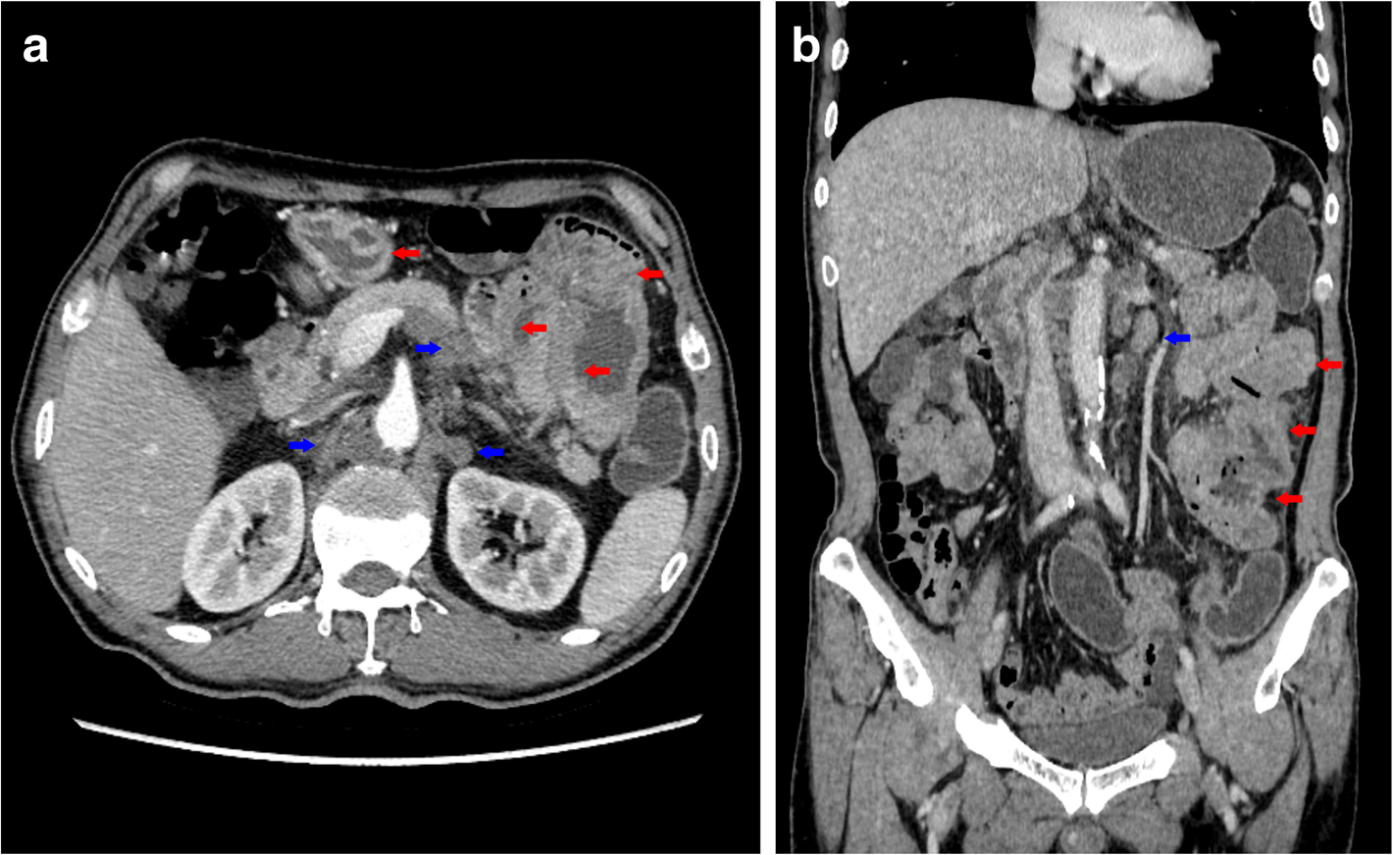
Abdominal CT. **a-b** Multiple thickened small intestinal walls (red arrow) in the left abdomen and multiple enlarged lymph nodes (blue arrow) in the surrounding and posterior peritoneum

One month later, the patient came to our hospital. Abdominal CT (Fig. 2a-b) showed multiple thickened small intestinal walls in the left abdomen and peripheral exudation. Multiple enlarged lymph nodes were identified in the surrounding and posterior peritoneum, some of which were fused. Due to the increasing levels of tumor makers (Ca125: 37.67 U/ml; Ca153: 129.5 U/ml; Ca211: 25.1 ng/ml) and CT examinations, an intestinal tumor could not be discounted. He underwent palliative segmental resection of the jejunum. At laparotomy, five mass were identified at the jejunum (15, 30, 50, 70 and 80 cm from the ligament of Treitz). The largest diameter was 5 cm. The small bowel mass was of the infiltrating stenosis type and invaded the serosa. Multiple enlarged lymph nodes observed in mesentery and blood vessel roots had fused into a mass. There was no evidence of intraperitoneal dissemination or parenteral metastasis.

Pathology (No.18–01519) revealed 5 poorly differentiated small bowel carcinomas; the size of which were 3 × 3 × 1.5 cm, 6 × 4 × 1.5 cm, 6 × 5 × 1.5 cm, 6 × 5 × 1.5 cm and 5 × 5 × 1.5 cm respectively. SBA invaded the intestinal serosa. The lymph nodes (8/11) were metastatic and the margins were negative. Tumor staging was T4N2M0 (IIIB). Immunohistochemistry: CK(+), CK7(+), CK20(−), Vimentin (+), S-100(−), MelanA (−), CD34(−), CD117(−), Dog-1 (−), ki67 positive rate 90%, AFP (+/−), NKX3.1 (−), p40 (−), CgA (−), HER-2 (2+), MPO (−), LCA (−), cdx-2 (−), NapsinA (−), TTF-1 (−). The patient began chemotherapy postoperatively for 1 month and accepted 4 cycles of treatment (Irinotecan + Teggio). Postoperation pulmonary tuberculosis was diagnosed from positive sputum smear tests and anti-spasm treatment was continued. Finally, the patient was diagnosed with multiple synchronous SBA and pulmonary tuberculosis. Six months later, the patient died from brain metastasis.

## Discussion and conclusions

SBA is rare and most tumors are single and located in the duodenum. SBA often develops with vague and non-specific gastrointestinal symptoms, including obscure bleeding, abdominal pain, nausea and vomiting, weight loss, diarrhea, and intestinal obstruction. As a result of relative infrequency and lack of clear symptoms, the diagnosis of SBA occurs at advanced stages; with − 40% of patients showing lymph node metastasis (stage III), and 35 to 40% with distant metastasis (stage IV) [5]. Multiple synchronous SBA is a unique type of SBA, with studies on these tumors sparse. Only few reports show that it is difficult to diagnose [6]. Whilst CT scans can detect the lesions, they only identify the thickening of multiple segmental small intestinal walls. This makes it difficult to distinguish multiple synchronous SBA from IBS. Our patient showed pulmonary tuberculosis and so intestinal tuberculosis was considered and the operation was delayed. Protocols to identify multiple synchronous SBA at an early stage are currently lacking and are urgently required.

Screening high-risk patients with typical symptoms is an option for early disease identification [7]. Chronic inflammation is associated with the formation of several malignant tumors, with Crohn’s disease for example increasing the risk of SBA [8]. Patients with familial adenomatous polyposis (FAP), hereditary non-polyposis colorectal cancer (HNPCC), Peutz-Jeghers syndrome, and celiac disease also show an increased risk of SBA development [9, 10]. To our knowledge, this is the first report of a patient with simultaneous multiple synchronous SBA and pulmonary tuberculosis. This is a rare case. The patient had both a rare tumor and an infectious disease. This complex condition made it easier for non-specialist physicians to perform an incorrect diagnosis. This misdiagnosis reminds us that: Firstly, we should not ignore multiple synchronous SBA when diagnosing intestinal diseases (especially IBS). Secondly, intestinal tuberculosis may represent risk factor for multiple SBA, although the pathology does not provide sufficient evidence.

To date, no specific methods exist for the diagnosis of multiple synchronous SBA. Examinations must be completed and we should obtain the pathology of high-risk patients as much as possible. An interesting issue from this case was how to identify SBA among at-risk patients. Crohn’s disease is typically accompanied by perianal abscesses, perianal fistula and extra intestinal manifestations. Intestinal tuberculosis presents as by pulmonary tuberculosis. Abdominal CT provides only limited diagnostic benefits and small bowel angiography is unsuitable for bowel obstructions. Multidetector row helical computed tomography [11], and magnetic resonance imaging [12] can facilitate the diagnosis of small bowel disease. Gastroscopy and colonoscopy should be performed, if the tumor is located close to the proximal duodenum or far from the terminal ileum. The remainder of the small bowel cannot be accessed without the use of video capsule endoscopy (CE) or double balloon enteroscopy (DBE). The definitive diagnostic yield of CE is as low as 20–30%, whilst DBE accounts for 60–70% of the diagnostic yield for intestinal disease [13]. However, CE is suitable for diagnosing scattered, small, or multiple lesions, in addition to active bleeding. The procedure is convenient, non-invasive, safe and comfortable. Doctors should strive to obtain pathology under endoscopy and avoid diagnostic treatments and treatment delays. In addition, the levels of Ca211 and other tumor markers including Ca125, Ca153 progressively increased in this case. For high-risk factors, such as FAP, elevated tumor markers and abnormal imaging examinations, laparotomy is recommended.

To-date, surgical resection remains the major treatment for patients with SBA. The 5-year survival is poor and dependent on tumor stage: 50–60% for stage I, 39–55% for stage II, 10–40% for stage III and 3–5% for stage IV [14]. The majority of diagnosis of SBA occurs at stage IV. This is because the disease is easily misdiagnosed and surgery is delayed. No dedicated TNM staging for multiple synchronous SBA exists. A higher number of adjuvant therapies are used for SBA treatment due to its poor prognosis and high risk of relapse [15, 16]. Chemotherapy is the main adjuvant strategy in patients with SBA. However, detailed chemotherapeutic regimens for SBA are lacking. The most common chemotherapy drug is fluorouracil but its benefits are modest. The patient had brain metastasis after 4 cycles of chemotherapy. Only the early diagnosis of multiple synchronous SBA with complete excision can improve long-term survival.

In this study, we highlighted how multiple synchronous SBA is rare and easily misdiagnosed during the early stages. In suspected cases, patients should receive complete tumor marker examinations, gastroscopy, enteroscopy or capsule endoscopy, and pathology under endoscopy. Active laparotomy should also be performed.

## Data Availability

All data and materials are available in this article.

## Abbreviations

SBA: Small bowel adenocarcinoma
CT: Computed Tomography
T-spot: *Mycobacterium tuberculosis* specific T lymphocyte
TB-IGRA: Interferon-gamma release assay for *Mycobacterium tuberculosis*
ESR: Erythrocyte sedimentation rate
ASO: Anti-streptolysin
RF: Rheumatoid factor
Ca125: Carbohydrate Antigen 125
Ca153: Carbohydrate Antigen 153
Ca211: Carbohydrate Antigen 211
SCC: Squamous cell carcinoma antigen
FAP: Familial adenomatous polyposis
HNPCC: Hereditary non-polyposis colorectal cancer
CE: Capsule endoscopy
DBE: Double balloon enteroscopy
AFP: Alpha fetoprotein
MPO: Mouse myeloperoxidase
LCA: Leukocyte common antigen
TTF-1: Thyroid transcription factor-1
HER-2: Human epidermalgrowth factor receptor-2
CK: Cytokeratin
CgA: Chromogranin A
IBS: Inflammatory bowel disease
